# How to Weigh Values in Value Sensitive Design: A Best Worst Method Approach for the Case of Smart Metering

**DOI:** 10.1007/s11948-019-00105-3

**Published:** 2019-04-08

**Authors:** Geerten van de Kaa, Jafar Rezaei, Behnam Taebi, Ibo van de Poel, Abhilash Kizhakenath

**Affiliations:** 1 Faculty of Technology, Policy, and Management, Delft University of Technology, Delft, The Netherlands; 2 KPMG, Zurich, Switzerland

**Keywords:** Best worst method, Technology acceptance, Smart metering, Value, Values

## Abstract

Proactively including the ethical and societal issues of new technologies could have a positive effect on their acceptance. These issues could be captured in terms of values. In the literature, the values stakeholders deem important for the development of technology have often been identified. However, the relative ranking of these values in relation to each other have not been studied often. The best worst method is proposed as a possible method to determine the weights of values, hence it is used in an evaluative fashion. The applicability of the method is tested by applying it to the case of smart meters, one of the main components of the smart grid. The importance of values is examined for three dimensions of acceptance namely sociopolitical, market, and household acceptance.

## Introduction

Technology development is value laden. That is, technological development and deployment can undermine or promote certain values (Van den Hoven 2005). Values-oriented approaches to design such as the Value Sensitive Design or the Design for Values approaches rest on the assumption that “explicit and transparent articulation of values” is highly relevant to design and innovation and it will “allow designing for shared public values” (Van den Hoven et al. 2015b, p. 3).

Value ladenness of technology is particularly intricate when it comes to complex technological systems such as electrical grids, in which different technological components and standards are dependent on each other (Suarez 2004; Ligtvoet et al. 2015). An example of such a system is the smart grid system which is an improved version of the electrical grid. The smart meter is one of its key components. This paper uses smart grids as the primary example to explicate the methodology used in this paper, whereby the focus lies on the acceptance of smart meters as their key component. The interdependence of the elements of complex systems requires cooperative activities as well as aligning the interests and values of the stakeholders belonging to the complex system’s technological and economic domains (Rosenkopf and Tushman 1998). However, stakeholders’ perspectives, understandings, and values within these domains may differ and this may result in conflicts (Ligtvoet et al. 2015). The smart meters’ mandatory rollout in the Netherlands is an case of how failing to include ethical issues (e.g. privacy) can result in societal rejection, which in this case ultimately postponed the smart meter’s rollout several years (Von Schomberg 2011; Ligtvoet et al. 2015). When complexity of the system increases, more stakeholders get involved requiring a better alignment of their opinions for society acceptance of the technology (Suarez 2004).

To better understand technology acceptance, a host of values need to be considered prior to and during the development and implementation of new technology. This is in line with the recent focus in the literature on responsible innovation that emphasizes a timely assessment and inclusion of social and ethical issues of new technology (Hellström 2003; Owen et al. 2013; Stilgoe et al. 2013; Van den Hoven et al. 2015b; Von Schomberg 2011). This paper builds on the notion that these social and ethical issues are best captured in *values* defined as “what a person or group of people consider important in life” (Friedman et al. 2013). Responsible innovation requires the timely and adequate inclusion of these values in the development of new technology (Taebi et al. 2014), assuming that these values represent the main social and ethical considerations. A responsible innovation of smart grids requires that the values at stake are identified and incorporated in the design to the extent possible. In this paper, values are investigated in relation to the acceptance of new technologies. Building on previous research (Taebi 2017: 1818), a distinction is made between social acceptance, which refers “to the fact that a new technology is accepted or merely tolerated by a community”, and ethical acceptability as “a reflection on a new technology that takes into account the moral issues that emerge from its introduction”. While acceptance and acceptability are somewhat related, discussing them separately allows to emphasize that they both deserve attention for the governance of technology. The literature tends to focus more on social acceptance whereas ethical acceptability remains underdeveloped. The values approach taken in this paper allows an examination of acceptability through this specific lens. This, however, does not imply that if a technology takes all values into account, it is automatically accepted in the society. This paper will investigate how including values (related to acceptability) could affect social acceptance.

Values Sensitive Design (VSD) and Design for Values (DfV) are two main approaches that aim to design the technology and its surrounding institutions for values. The VSD has primarily been applied to information technology and, specifically, to human computer interaction (Friedman 1997; Friedman et al. 2002; Flanagan et al. 2008; Friedman and Kahn 2000; Van den Hoven 2007), but the approach has later been expanded to address inclusion of moral values in other technological domains (Brey 2014; Umbrello 2018; Umbrello and De Bellis 2018; Vermaas et al. 2010; Van den Hoven et al. 2012). Scholars of VSD argue that the design process has value implications because new technology can shape our practice and hence undermine or promote certain values (Van den Hoven 2005). The Design for Values (DfV) approach furthers this discussion and aims to explicitly and transparently articulate values in design and innovation such that it will “allow designing for shared public values” (Van den Hoven et al. 2015b).

Indeed, values need to be compared and ranked, especially when non-compatible values point in different directions for the development of new technologies. That is, when one runs into value conflicts (Van de Poel 2009, p. 977). In these circumstances, two scenarios are possible: “(i) changing the design in such a way that it accommodates these conflicting values or (ii) making a value trade-off that decides which value should take priority in the design” (Taebi et al. 2014). Van de Poel (2015a) presents several approaches for systematically dealing with conflicting values in Van de Poel (2015a). One of these methods, for instance, is the Satisficing approach, which involves trading off the loss of one value in terms of a gain in a different value—satisficing, or *satisfying* a threshold (*sufficient*) for each value, and trading off only the *surplus* value above the threshold.

More recently, scholars have presented a novel approach in which value profiles of groups of actors are created and subsequently translated into design requirements following the principles of constructive dialogue and the constant-sum approach (Flipse and Puylaert 2017). Other subjective methods include the Q-sort method (Block 1961) and the Delphi method (Linstone and Turoff 1975), which are used to rank values more qualitatively. Other methods elicit the weights based on the preferences of experts/users/decision-makers. These include Simple Multi-Attribute Rating Technique (SMART), Swing (Edwards and Barron 1994; Mustajoki et al. 2005; Edwards 1977), Analytic Hierarchy Process (AHP) (Saaty 1977), Analytic Network Process (ANP) (Saaty 1990), and Best worst method (BWM), which has gained enormous attention since its introduction (Rezaei 2015). The BWM has been applied to many real-world decision-making problems because (1) it is a very structured method, which helps the decision-maker/evaluator provide more reliable inputs; (2) it uses the pairwise comparison in an efficient, yet sufficient, way, which attracts the involvement of the decision-makers; (3) the outputs have been found more reliable compared to other methods such as AHP; (4) it is a suitable method for determining the weights of the criteria when there is a set of qualitative and quantitative criteria; and (5) it has a particular metric to check the reliability of the final outputs.

The BWM facilitates a comparison based on relative importance of values. The BWM is a stand-alone method, but it can also be considered as an extension to the Satisficing approach. That is, sometimes it may be necessary to set a threshold to protect certain values, for instance, because of legally required or otherwise obligated codes and standards (e.g. Van de Poel 2009). The Satisficing approach does not specify how trade-offs are facilitated above the thresholds, whereas the BWM does.

To devise effective strategies for governing complex systems such as smart grids, it is necessary to understand the relative importance of different values from stakeholders’ perspectives. To this end, the main objective of this paper is: to propose the BWM (Rezaei 2015, 2016) as a weight elicitation method in order to determine the weights of values with regard to each other. The importance of the values for each of the dimensions of technology acceptance for the case of smart metering is investigated with the aim of testing the applicability of the method, a unique contribution of this paper.

In this paper it is assumed that the identified values, and more specifically their relative ranking, determine the acceptance of new technology. Thus, in this paper, a different position towards technology acceptance is presented that relates more to values prioritization than how single value are conceived. It is particularly this relative relevance of values that gives rise to various societal and methodological challenges (i.e. when considered in isolation many values can be found to be important). But when choices among the values have to be made, intricate questions will arise regarding the importance of one value, when considered in conjunction or in conflict with another value. Technological design often poses these situations in which choices must be made between important values at stake. A second assumption which is made in this paper is that ethical acceptability can be captured in terms of values.

## Social Acceptance, Ethical Acceptability, and Values

Ethics of technology scholars have focused on the questions of what values are and why they are important in technological developments. It has been argued that technological design may incorporate certain values, since new technologies can promote or undermine certain values (Van de Poel 2009; Van den Hoven et al. 2015a; Taebi and Kloosterman 2015). If values are not appropriately included in the design or implementation of a technology, it could lead to controversies and ultimately to the failure of such projects (Dignum et al. 2016; Pesch et al. 2017). The literature on responsible innovation is based on this rationale, namely, that a lack of proper (and timely) inclusion of societal and ethical issues in technological developments could lead to the non-acceptance of technological innovations. Although it cannot be argued that including these issues would *ensure* acceptance, it would seem reasonable to assume that it could increase the likelihood of acceptance. The focus of this paper is not on how to increase the acceptance of new technologies but to investigate whether there is a link between acceptance and a proper inclusion of the values at stake.

Take for example the smart meter technology. Some scholars focus on factors that affect the implementation of smart meters among consumers (Chen et al. 2017; Chou and Yutami 2014), whereas other researchers study smart meters in relation to technology acceptance. The latter group mostly agree that the implementation of smart meters will lead to privacy concerns (Cuijpers and Koops 2013; King and Jessen 2014). Such concerns could be addressed through technological solutions and policy interventions. For example, some scholars have discussed various ‘privacy friendly techniques’ that minimize or avoid the use of personal data (McKenna et al. 2012) whereas others focus on policy interventions such as mandatory versus voluntary roll-out targets or privacy and data security policies (Zhou and Brown 2017). Recently, scholars have argued that a broader range of moral values beyond privacy—i.e. trust, inclusiveness, and affordability of energy—can influence the social acceptance of smart grid technology (such as smart meters) (Milchram et al. 2018). What these and other studies have in common is that they implicitly argue that incorporating social and ethical values (mostly privacy) into smart meters may increase the likelihood of their acceptance. For example, it has been shown that if the privacy issues associated with smart meters had been more appropriately included in the design and during the implementation of smart meters in the Netherlands, these smart meters would have had a higher likelihood of acceptance among stakeholders (Ligtvoet et al. 2015). Although various studies [e.g. Van den Hoven et al. (2015a)] have indicated the importance of paying attention to values when designing technologies, these studies have not focused on whether it is possible to assign a weight to each value. The current study focuses on this.

Various scholars in the field of ethics of technology distinguish between social acceptance and ethical acceptability (Oosterlaken 2015; Taebi 2017; Van de Poel 2016; Doorn and Taebi 2018). Whereas scholars with a background in the social sciences refer to the (social) acceptance of technology, philosophers have long focused on its ethical acceptability, which is reflected in values (Taebi 2017). As mentioned in the introduction, acceptance refers “to the fact that a new technology is accepted or merely tolerated by a community”—and ethical acceptability refers to “a reflection on a new technology that takes into account the moral issues that emerge from its introduction” (Taebi 2017: 1818). In this paper, acceptability is embodied in terms of values. In other words, acceptance and acceptability are interconnected. It is argued that there is a gap between ethical acceptability and social acceptance and that this gap needs to be bridged (Taebi 2017). Social acceptance studies are not capable of addressing all the relevant ethical issues associated with technology, while philosophical analyses are often conceptual and lack stakeholders’ opinions (Taebi 2017). One way to bridge the gap is to conceive acceptability as acceptance under ideal reasoning conditions, or to extend acceptance studies to values that are ethically important even if they are not explicitly articulated by stakeholders. The latter approach is taken in this paper.

The paper focuses on the acceptance of renewable energies and, following earlier research, defines three dimensions of acceptance (Wüstenhagen et al. 2007a). Sociopolitical acceptance is “social acceptance on the broadest, most general level”, market acceptance is “the process of market adoption of an innovation”, and community acceptance is defined as the specific acceptance of new proposed technology by stakeholders. This division is similar to a technological artefact’s design process, where its value is perceived differently depending upon the stakeholder groups that are involved in the process in which value for the artefact is created (Kroes and Van de Poel 2015).

*Sociopolitical acceptance* focuses on policies for the technology and its acceptance and on the key stakeholders’ trust (e.g. market actors and end users) (Wüstenhagen et al. 2007a). To design policies that are appropriate it is needed to adhere to technological criteria and to the preferences and demands of end users (Chou and Yutami 2014). Many studies argue that technological systems are not value neutral, but that they inherently embrace certain values (and exclude others). For instance, technological systems might consistently favor the interests of economically and politically powerful people (Friedman and Kahn Jr. 2002).

The key determinant of *market acceptance* is the possession of an installed base. Innovation management and standardization scholars have offered factors that may positively influence installed base and technology commercialization (Schilling 1998, 2002; Suarez 2004; Van de Kaa et al. 2011, 2015; Van de Kaa and De Bruijn 2015) and have assigned weights to these factors (Van de Kaa et al. 2014, 2018). These are important for grid operators (DNOs), energy suppliers, home energy management systems (HEMS), and for suppliers and vendors of smart meters. These stakeholders will have to make a decision which technology to support and adopt, and insights about the importance of factors that affect market acceptance of the technologies may decrease the uncertainty that is attached to that decision.

*Community acceptance* is crucial for siting decisions about renewable projects (Wüstenhagen et al. 2007b). Since smart meters require acceptance by individual households, this dimension is renamed to *household acceptance*. As argued by Ligtvoet et al. (2015), households “may have a less prominent role in determining the development of technologies, but at times play a significant role in the acceptance of the technology” (Mitchell et al. 1997). Technology acceptance can be increased by meeting both the functional and end users’ moral and social values.

## Developing a Methodology for Assigning Weights to Values

Multiple values need to be considered when evaluating technology acceptance. This implies that the problem can be formulated as a multi-criteria decision analysis, as values can be considered as criteria. There are different multi-criteria decision analysis methods in the existing literature (for the most commonly used methods see, for example, Figueira et al. (2005). In this study one of the most recently developed multi-criteria decision analysis methods, called the BWM, is used. This method is chosen as it has been shown to be a more reliable weighting method compared to matrix-based methods such as AHP, which needs fewer data compared to similar methods. This method allows you to assign weights for each value in a dimension, showing the importance of the values in each dimension. BWM is applied to determine weights for values for the three conceptualizations of technology acceptance. The methodology consists of two steps: (1) relevant values are chosen, (2) the BWM approach is applied.

## Choosing Relevant Values

To identify the relevant values, the literature on technology acceptance for smart metering is first reviewed to examine the values that may have played a role. Three key papers are selected. First, a key paper by Wüstenhagen et al. (2007a) that discusses sociopolitical acceptance is selected. This paper is considered to be a representative paper from the energy policy literature. Second, an article by Van de Kaa et al. (2011) is selected. This paper lists the most complete set of factors for the market acceptance of a technology. This list of values mostly comes from the innovation management literature (Suarez 2004). Third, a paper by AlAbdulkarim and Lukszo (2011) is selected. This paper discusses household acceptance and addresses the acceptance of smart meters in the Netherlands from a user perspective. Their list of values mostly comes from the value sensitive design (VSD) literature (Friedman and Kahn Jr. 2002) regarding moral and social values and functional values for end-users of smart meters. For each of these three papers, a forward (papers that cited the key paper) and a backwards literature search (references mentioned in the key paper) using the search engines ISI Web of Knowledge and Scopus was performed. Each paper was carefully read and it was analysed whether a value was mentioned in the paper. A value could be mentioned explicitly or implicitly. If so, it was included in the list of relevant values. This process was continued until the repetitions of the arguments indicated that a sufficient overview of values had been achieved.

As several values have been proposed in the existing literature, a qualitative validation was first conducted and the list of values was screened by interviewing a panel of experts with extensive knowledge on dimensions of acceptance. Three researchers were selected that focus on either smart grid and/or smart meters. They were chosen based on their level of expertise on smart meters and smart grid systems. They all had four or more years of experience in the development and deployment of the smart meter and smart grid systems. A qualitative validation guarantees that the range of values applies to the framework for the social acceptance of smart meters (Kheybaria et al. 2019; Van de Kaa et al. 2018). One expert was an advisor in the market, as well as an end user (representative of a household) and had experience with the institutional design of smart meters. The second expert was a researcher on smart meters and smart grid systems, focusing on consumer behavior. The third expert was a researcher on the social acceptance of energy projects.

By qualitatively validating the values with these experts, their views and preferences about the values could be assessed and by comparing their views and preferences, a clear set of values could be determined for a dimension or group of stakeholders that was needed to compare the values’ importance (see Tables 1, 2, 3).

**Table 1:** Values for sociopolitical acceptance

Value	Definition
Privacy	To ensure privacy, the private space of end users should be kept free from intrusion and users should be allowed to determine what information about themselves can be communicated (Friedman et al. 2013; Warnier et al. 2015)
Environmental sustainability	To ensure environmental sustainability, energy consumption should not burden the environment (Friedman et al. 2013; Taebi and Kadak 2010)
Compatibility	To ensure compatibility, the technology should adequately perform its function in conjunction with other apparatus (similar products and complementary devices) and the infrastructure (Van de Poel 2015b)
Cost-effectiveness	To ensure affordability and its continuation over the course of time (Taebi and Kadak 2010)
Trust	To promote trust and expectation that exists between the people (actors) who can experience good will, extend good will toward others, feel vulnerable, and experience betrayal (Friedman et al. 2013; Huldtgren 2015)
Reliability	To ensure reliability and to perform without failing and without grid malfunctioning (i.e. blackouts) (Van de Poel 2015b)
Autonomy	To ensure autonomy so that users have control over the technology to plan and execute their actions in way to achieve their goals (Friedman et al. 2013; Warnier et al. 2015)
Procedural justice	To ensure transparency, honesty and as well as timely, full, and unbiased information in decision-making (Dignum et al. 2016)

**Table 2:** Values for the market acceptance of smart meters

Value	Definition
Efficiency	To ensure efficiency, the technology’s ratio between the degree to which it fulfil its function and the effort (data-rate, latency, rang etc.) to achieve that effect should be optimized (Erlinghagen et al. 2015; Van de Poel 2015b)
Reliability	To ensure reliability and to perform without failing and without grid malfunctioning (i.e. blackouts) (Van de Poel 2015b)
Compatibility	To ensure compatibility, the technology should adequately perform its function in conjunction with other apparatus (similar products and complementary devices) and the infrastructure (Van de Poel 2015b)
Flexibility	To ensure flexibility and to adapt to changes in customer needs and new technological developments (Van de Kaa et al. 2011)
Procedural justice	To ensure transparency, honesty and as well as timely, full, and unbiased information in decision-making (Dignum et al. 2016)
Ownership	To ensure resources and competences for the communication network for smart meters, to use it, to manage it, and to derive income from it (Huldtgren 2015; Erlinghagen et al. 2015)
Cost-effectiveness	To ensure affordability and its continuation over the course of time (Taebi and Kadak 2010)
Disclosure	To ensure that accurate information about the benefits and harms of the technology is provided (Friedman et al. 2000)
Trust	To promote trust and expectation between the people (actors) who can experience good will, extend good will toward others, feel vulnerable, and experience betrayal (Friedman et al. 2013; Huldtgren 2015)

**Table 3:** Values for the household acceptance of smart meters

Value	Definition
Security/safety	To ensure protection from intentional harmful attacks (e.g. cyber-attack, burglary) and unintentional effects (loss of user-data) (Taebi and Kadak 2010)
Usability	To ensure that every household can successfully use the smart meter and its functionalities (Huldtgren 2015)
Comfort	To provide advanced technology to control and manage electricity use (Gangale et al. 2013) and to offer technological solutions allowing the optimization of comfort and more control over own energy use
Cost-effectiveness	To ensure that affordability regarding cost and benefit, when choosing for the technology can be guaranteed and to ensure its continuation over the course of time (Erlinghagen et al. 2015; Taebi and Kadak 2010)
Trust	To promote trust and expectation that exists between the people (actors) who can experience good will, extend good will toward others, feel vulnerable, and experience betrayal (Friedman et al. 2013; Huldtgren 2015)
Privacy	To ensure privacy, the private space of end users should be kept free from intrusion, and users should be allowed to determine what information about themselves can be communicated (Friedman et al. 2013)
Autonomy	To ensure autonomy so that users have control over the technology to plan and execute their actions in way to achieve their goals (Friedman et al. 2013)
Distributive justice	To ensure distributive justice, the distribution of the cost and benefits and other positive and negative effect of the technology should be fair (Dignum et al. 2016)
Environmental sustainability	To ensure environmental sustainability, energy consumption should not burden the environment (Friedman et al. 2013; Taebi and Kadak 2010)

## BWM

After selecting the most relevant values using the above approaches, the BWM was used to assign weights to these values. Ten experts evaluated the importance of the values for the acceptance of smart metering with the help of the BWM. These experts were asked to empathize with the general public and they were asked what the general public would think about certain matters. Information about the ten experts’ background and years of experience with smart meters and smart grid technology can be found in Table 4. The BWM will now be described in more detail.

**Table 4:** Background of experts

Expert	Field of expertise	Years of experience
1	Advisor and developer of smart meters. End user with home energy management system (HEMS)	> 10
2	Innovation manager at DNO. Involved in implementation project of smart meters	6
3	PhD candidate. Focus on demand side management stakeholder analysis	4
4	PhD candidate. Focus on implementation and regulation for smart meters and demand response	5
5	PhD candidate. Focus on customer behavior of smart meter customers	3
6	Advisor and previous employee at a DNO. Involved in customer center and meetings with the Ministry of Economic Affairs	6
7	Strategy manager at DNO. Advisor and developer of the smart meter policies in Europe	> 10
8	Strategy manager at DNO. Advisor and developer of smart meter systems	10
9	HEMS and energy management service provider. Previous advisor for utility firm. Expert on customer behavior for energy usage in households	6
10	Advisor and consultant. Focus on data management systems of smart meters and HEMS customers	8

The BWM is a multi-criteria decision analysis method, which has been shown to be a suitable methodology to structure highly complex systems so that decision-makers and policymakers would be able to better understand the system of interest. The method has been applied in many different areas including transportation (Groenendijk et al. 2018; Rezaei et al. 2018b), energy (Omrani et al. 2018; Zhao et al. 2018; van de Kaa et al. 2019a, b; Kheybaria et al. 2019), sustainability (Rezaei et al. 2019; Garg and Sharma 2018; Nie et al. 2018), supply chain and logistics (Lo et al. 2018; Rezaei et al. 2015, 2016, 2019; Ahmad et al. 2017; Ahmadi et al. 2017; Haeri and Rezaei 2019; Onstein et al. 2019; Liu et al. 2019), ICT (Nawaz et al. 2018; Van de Kaa et al. 2018), water (Nie et al. 2018), aviation (Rezaei et al. 2018a), research assessment (Salimi 2017), and R&D (Salimi and Rezaei 2018).

This method helps decision-makers to assess the importance of the factors constituting the overall output(s) of the system quantitatively (Rezaei et al. 2015). In other words, it is a suitable method to evaluate a complex system when several qualitative and quantitative factors/criteria play a role. The BWM is based on pairwise comparison, in which two values are compared. In practice, comparing values is a challenge, especially when it comes to making consistent comparisons between all values. Building on earlier work (Rezaei 2015), it can be argued that it is easier for a decision-maker (or an expert) to express the direction of comparison than to rate the strength, which is a difficult task and usually the main source of inconsistency. The direction defines whether a value is more or less significant than the other, while the strength enumerates how much one value is more significant when compared to the other. The pairwise comparison of BWM solves this difficulty. Rather than comparing each pair of values, first the most important value (which is called the *best*) and the least important value (which is called the *worst*) of each dimension (set of values) are determined. The rest of the values from a particular dimension are then compared based on this reference value.

To determine the weights of the values (which are called criteria here) in a dimension (set of values) five steps are followed (Rezaei 2015). The method is depicted for the values of sociopolitical acceptance (*s*_1_, *s*_2_, …, *s*_*n*_):

*Step 1* The expert (or decision-maker or user) determines the values (criteria) relevant for the sociopolitical dimension of acceptance (*s*_*1*_, *s*_*2*_, …, *s*_*n*_).

*Step 2* The expert (or decision maker or user) determines the best (most important) and the worst (least important) values of this dimension of acceptance from a sociopolitical set of values (criteria) (*s*_*1*_, *s*_*2*_, …, *s*_*n*_).

*Step 3* The expert’s (or decision-maker or user) preferences of the most important value from the sociopolitical dimension are compared to the other values of this dimension, using a number between 1 (equally important) and 9 (extremely more important). These comparisons result in a best-to-others vector i.e. *S*_*B*_ = (*s*_*B*1_, *s*_*B*2_, …, *s*_*Bn*_) where *s*_*Bj*_ indicates the expert’s (or decision-maker or user) preference of the most important value *B* over value *j* of the sociopolitical dimension.

*Step 4* Similarly, the expert’s (or decision-maker or user) preferences of all the other values of the sociopolitical dimension are compared to the expert’s least important value of this dimension using a number between 1 and 9. These comparisons result in an others-to-worst vector i.e. *S*_*W*_ = (*s*_1*W*_, *s*_2*W*_, …, *s*_*nW*_)^T^ where *s*_*jW*_ indicates the preference of the expert value *j* over the least important value *W* of the sociopolitical dimension.

*Step 5* The last step focuses on deriving the optimal weights (importance) for each value (*w*_*s*1_, *w*_*s*1_, …, *w*_*sn*_). A solution can be found when the maximum absolute difference for all *j* is minimized for the following set $\left\{ {\left| {w_{SB} - s_{Bj} w_{sj} } \right|,\left| {w_{sj} - s_{jW} w_{SW} } \right|} \right\}$ (Rezaei 2016), which results in the following optimization problem.

1 $$\begin{aligned} & minmax_{j} \left\{ {\left| {w_{SB} - s_{Bj} w_{sj} } \right|,\left| {w_{sj} - s_{jw} w_{SW} } \right|} \right\} \\ & {\text{subject}}\;{\text{to}} \\ & \mathop \sum \limits_{j} w_{sj} = 1 \\ & w_{sj} \ge 0\;{\text{for}}\;{\text{all}}\;j \\ \end{aligned}$$

This formulation can be translated to a linear programing problem as follows:

2 $$\begin{aligned} & min\xi^{L} \\ & \left| {w_{SB} - s_{Bj} w_{sj} } \right| \le \xi^{L} ,{\text{for}}\;{\text{all}}\;j \\ & \left| {w_{sj} - s_{jw} w_{SW} } \right| \le \xi^{L} ,{\text{for}}\;{\text{all}}\;j \\ & \mathop \sum \limits_{j} w_{sj} = 1 \\ & w_{sj} \ge 0,{\text{for}}\;{\text{all}}\;j \\ \end{aligned}$$

Model (2) is a linear programming problem which can be solved by simple optimization tools. The solution to this model is the weights of the values $(w_{S1}^{*} , w_{S2}^{*} , \ldots , w_{Sn}^{*} )$, in this case for sociopolitical acceptance. For the linear model of BWM, $\xi^{L*}$ is considered as a consistency indicator of the comparison system and values of $\xi^{L*}$ closer to zero show a higher level of consistency (Rezaei 2016). The steps are presented for the sociopolitical acceptance dimension. The weights for the other dimensions, market acceptance $(w_{M1}^{*} , w_{M2}^{*} , \ldots , w_{Mn}^{*} )$ and household acceptance $(w_{H1}^{*} , w_{H2}^{*} , \ldots , w_{Hn}^{*} )$ are derived following the same steps.

## Results

The results of the BWM for the importance of values for sociopolitical acceptance, market acceptance, and household acceptance of smart meters are shown in Table 5. The results of the weights of the values were accumulated and the average was calculated. The average consistency indicator $\xi^{L*}$ of comparison systems is included. The comparisons had high consistency, since the consistency ratio is very low (< 0.06) and closer to zero than one.

**Table 5:** Average weight for the importance of the values for the three conceptualizations of acceptance of smart meters

Sociopolitical acceptance	Weight	Market acceptance	Weight	Household acceptance	Weight
Privacy	0.176	Cost-effectiveness	0.159	Privacy	0.157
Environmental sustainability	0.150	Reliability	0.134	Security/safety	0.147
Procedural justice	0.146	Efficiency	0.129	Usability	0.135
Reliability	0.121	Compatibility	0.124	Comfort	0.127
Cost-effectiveness	0.113	Procedural justice	0.112	Trust	0.111
Trust	0.110	Trust	0.095	Autonomy	0.104
Compatibility	0.093	Flexibility	0.0834	Cost-effectiveness	0.092
Autonomy	0.091	Ownership	0.0830	Environmental sustainability	0.080
		Disclosure	0.079	Distributive justice	0.048
consistency $\xi^{L*}$	0.056	Consistency $\xi^{L*}$	0.052	Consistency $\xi^{L*}$	0.053

The results of the BWM show that, according to the experts, privacy is the most important value for achieving sociopolitical acceptance and household acceptance and cost-effectiveness is the most important factor for achieving market acceptance. Indeed, Ligtvoet et al. (2015) has hinted towards the importance of guaranteeing privacy for smart meters and also explained why privacy incorporation is so important. Our study provides the first hard evidence of the importance of guaranteeing privacy to ensure technology acceptance. The results also show that cost-effectiveness is the most important factor for achieving market acceptance. In other words, the installation of the smart meter should be affordable for potential adopters. These costs should outweigh the benefits. Indeed, in the Netherlands, most households have a regular meter. Changing to a smart meter costs about 80 euros. The benefits of a smart meter include automatic communication of energy consumption data to the energy companies and a better overview of their energy consumption for customers. Eventually this could lead to savings, but the initial investment of 80 euros seems to be a hurdle for many consumers. One solution could be to apply a form of penetration pricing whereby the price of the product is considerably lower than the actual costs to produce the product. This can quickly lead to a large market share and thus to market acceptance (Adams 1996; Katz and Shapiro 1986). Indeed, in the Netherlands the smart meter is installed for free in the period 2015–2020. Since cost-effectiveness is so important for reaching market acceptance, we believe that this will increase the chances that the smart meter will eventually become adopted on a large scale.

## Discussion

This paper contributes to the literature by providing a methodology that can rank values based on expert opinions. It is important to keep in mind that there are differences between expert-based methods (such as the one used in this study), and data-based methods (such as most statistical analysis) in terms of the amount of data needed for reliable results. In statistical analysis, data is usually collected from a large sample of observations to find something from the sample and get some ideas about the larger population, usually with the purpose of generalizations. In expert-based methods, one relies on the knowledge and opinions of a limited number of experts. In other words, the choice of experts is of utmost importance. At first glance, including only ten experts in the study might seem insufficient. However, the aim of this study is not to propose a recommendation for policy. The aim is to show that the BWM can effectively establish the relative ranking of values, which can be helpful when dealing with value conflicts. To that end, the paper has provided evidence that the BWM can be used to better understand the importance of values for smart meter acceptance and can help to get a first indication of weights for values for the case of smart metering. Expert opinions have been included here to show how this method could work, also when applied to broader groups of people.

The methodology has some limitations. First, the paper assumes that acceptance is determined by stakeholders’ perspectives on acceptance, which in turn are influenced by whether stakeholders’ values are sufficiently considered in the design. However, the question is whether single experts can sufficiently represent a group of people (such as households) and whether they can take into account the values of others. Each individual stakeholder in a group (dimension) can have different sets of values, which are important for the decision to accept a smart meter. Assessing the opinion of each individual and aggregating their judgments would be impossible. And there is a fundamental objection against this from an ethical perspective, since an aggregation of individual values does not necessarily constitute broader shared or societal values. Nevertheless, future research that aims to use the methodology could assess the acceptance of smart meters in each category of acceptance in more detail by further segmentation. For example, household acceptance refers to all the smart meter customers holding the same values. This segmentation could give information about which values are shared in a certain segment. Scholars have suggested that end user groups should be further segmented, and have identified four smart meter customer groups for the Swiss market (Kaufmann et al. 2013). Experts who evaluated the values also called attention to the idea that it might be possible to further segment household acceptance into different group interests, such as price sensitive, technically skilled or end users who want autonomy of the network. These and future segmentations will provide information that can lead to specific values for smart meter acceptance, which can be used to offer different service options for each end user segment. In a follow-up study, further segmentation could provide insights and design requirements for specific services of smart meters and could foster more customer involvement. In conclusion, although the method could be expanded to include the opinions of various groups of stakeholders, challenges are likely to arise when applied to a broader group of individuals. These include how to deal with diverging opinions that represent different interests and whose opinion to include in the analysis. However, these challenges are general for all methods that aim at including stakeholders’ values in the analysis [see for example Taebi et al. (2014)].

Another limitation is that the paper has assumed trade-offs between the values that cannot be achieved simultaneously. Philosophical and psychological literature suggests that trade-offs between values are sometimes considered taboo by people, or that trade-offs are impossible because values are incommensurable (Chang 1997; Raz 1986; Tetlock 2003; Van de Poel 2015a). It has been suggested that in such cases, values may be constraints on what options (technologies) are acceptable, rather than being criteria that can be traded off (Van de Poel 2015a). If values indeed act as constraints in such cases, it might be possible to select acceptable options by first leaving out the options that do not meet certain constraints derived from the relevant values, and then applying a multi-criteria decision method like the BWM. This resembles the Satisficing method as discussed in the Introduction, in that certain values will then have to be met completely and are positioned outside the trade-off zone. In this sense, the BWM could be considered as a method that complements the earlier mentioned methods in the literature.

Finally, two remarks are in order. Firstly, the paper does not claim that a quantitative analysis should completely replace a qualitative analysis. Rather, a quantitative analysis alludes to the direction in which a qualitative assessment should be made. For this paper, it helps to identify the most important values for dimensions of acceptance. Qualitative assessments have been presented in the first place as a response to quantitative mostly aggregate methods such as cost–benefit analysis, which have also been proposed in the literature for dealing for value conflicts (Van de Poel 2015a). This type of analysis has been criticized for wrongly eliminating all the complexity in decision-making by reducing all values into one single value of utility, which is then expressed in monetary terms (Hansson 2007). Rather than brushing aside all the complexity, the BWM method aims at clarifying the choices by hinting in the direction of how stakeholders are inclined to make such choices. Of course, this is just the starting point of the discussion on a comprehensive qualitative analysis. Secondly, the BWM has been shown to be a suitable methodology to structure highly complex systems for decision-makers and policymakers. This method is applied to assess the opinion of experts and as such it should be considered as a *proof of concept* and as the first step towards a comprehensive assessment of values in smart metering.

## Conclusion

This paper investigates the relation between social acceptance and ethical acceptability of new technologies. It is assumed that the ethical acceptability could be sought in the realm of values at stake. This paper’s case study was the acceptance of smart metering, in which the values that stakeholders found important were investigated in their development and introduction. Indeed, sometimes values cannot be achieved at the same time, which gives rise to a value conflict. For appropriate decision-making on new technologies issues, it is important to properly understand the multitude of values and also the potential conflicts that could arise between these values. In ethics of technology, a number of methods for dealing with value conflicts have been proposed. The proposed method in this paper fits well in the existing methods and complements different methods, all of which could facilitate a better and more informed way of dealing with conflicts. The method is, therefore, potentially relevant for both decision-makers and policymakers, since such an analysis can assist them in better understanding the system, and most importantly, anticipating conflicts and finding responsible approaches to resolve such conflicts. Expanded versions of what has been shown here could facilitate actual policy-making. It has been shown how analytical hierarchies between these sometimes-conflicting values can be operationalized. The paper provides a first indication that the BWM is a proper methodology for establishing the relative relevance of values.

The first assumption that was made in this paper refers to the notion that values, and more specifically their relative ranking, determine the acceptance of new technology. That assumption could be empirically tested in future research. Also, the second assumption which was made in this paper referring to the notion that ethical acceptability can be captured in terms of values could be empirically and conceptually tested in future studies. Can all ethical concerns of stakeholders, but also the broader concerns that are not directly linked to existing stakeholders (e.g. intergenerational justice) be captured in terms of values?

## References

[CR1] Adams M (1996). Norms, standards, rights. European Journal of Political Economy.

[CR2] Ahmad WNKW, Rezaei J, Sadaghiani S, Tavasszy LA (2017). Evaluation of the external forces affecting the sustainability of oil and gas supply chain using Best Worst Method. Journal of Cleaner Production.

[CR3] Ahmadi HB, Kusi-Sarpong S, Rezaei J (2017). Assessing the social sustainability of supply chains using Best Worst Method. Resources, Conservation and Recycling.

[CR4] AlAbdulkarim, L., & Lukszo, Z. (2011). Impact of privacy concerns on consumers’ acceptance of smart metering in the Netherlands. In *International conference on networking, sensing and control, Delft, 2011* (pp. 287–292).

[CR5] Block J (1961). The Q-sort method in personality assessment and psychiatric research.

[CR6] Brey PAE, Van den Hoven J, Vermaas PE, Van de Poel I (2014). Design for the value of human well-being. Handbook of ethics, values, and technological design.

[CR7] Chang R (1997). Incommensurability, incomparability, and practical reasoning.

[CR8] Chen C, Xu X, Arpan L (2017). Between the technology acceptance model and sustainable energy technology acceptance model: Investigating smart meter acceptance in the United States. Energy Research and Social Science.

[CR9] Chou J-S, Yutami GAN (2014). Smart meter adoption and deployment strategy for residential buildings in Indonesia. Applied Energy.

[CR10] Cuijpers C, Koops B-J, Gutwirth S, Leenes R, de Hert P, Poullet Y (2013). Smart metering and privacy in Europe: Lessons from the Dutch case. European data protection: Coming of age.

[CR11] Dignum M, Correlje A, Cuppen E, Pesch U, Taebi B (2016). Contested technologies and design for values: The Case of shale gas. Science and Engineering Ethics.

[CR12] Doorn N, Taebi B (2018). “Rawls’ wide reflective equilibrium as a method for engaged interdisciplinary collaboration: Potentials and limitations for the context of technological risks. Science, Technology and Human Values.

[CR13] Edwards W (1977). How to use multiattribute utility measurement for social decisionmaking. IEEE Transactions on Systems, Man, and Cybernetics.

[CR14] Edwards W, Barron FH (1994). SMARTS and SMARTER: Improved simple methods for multiattribute utility measurement. Organizational Behavior and Human Decision Processes.

[CR15] Erlinghagen S, Lichtensteiger B, Markard J (2015). Smart meter communication standards in Europe: A comparison. Renewable and Sustainable Energy Reviews.

[CR16] Figueira J, Greco S, Ehrgott M (2005). Multiple criteria decision analysis: State of the art surveys.

[CR17] Flanagan M, Howe DC, Nissenbaum H, Van den Hoven J, Weckert J (2008). Embodying values in technology: Theory and practice. Information technology and moral philosophy.

[CR18] Flipse SM, Puylaert S (2017). Organizing a collaborative development of technological design requirements using a constructive dialogue on value profiles: A case in automated vehicle development. Science and Engineering Ethics.

[CR19] Friedman B (1997). Human values and the design of computer technology.

[CR20] Friedman, B., Felten, E., & Millett, L. I. (2000). Informed consent online: A conceptual model and design principles. University of Washington Computer Science & Engineering Technical Report 00-12-2.

[CR21] Friedman, B., & Kahn, P. H. (2000). New directions: A value-sensitive design approach to augmented reality. http://citeseerx.ist.psu.edu/viewdoc/download?doi=10.1.1.102.5812&rep=rep1&type=pdf.

[CR22] Friedman B, Kahn PH, Sears A, Jacko JA (2002). Human values, ethics, and design. The human-computer interaction handbook.

[CR23] Friedman, B., Kahn, P. H., & Borning, A. (2002). Value sensitive design: Theory and methods. UW CSE technical report (pp. 1–8).

[CR24] Friedman B, Kahn PH, Borning A, Huldtgren A (2013). Value sensitive design and information systems (early engagement and new technologies: Opening up the laboratory).

[CR25] Gangale F, Mengolini A, Onyeji I (2013). Consumer engagement: An insight from smart grid projects in Europe. Energy Policy.

[CR26] Garg, C. P., & Sharma, A. (2018). Sustainable outsourcing partner selection and evaluation using an integrated BWM–VIKOR framework. *Environment, Development and Sustainability*, 1–29. 10.1007/s10668-018-0261-5.

[CR27] Groenendijk L, Rezaei J, Correia G (2018). Incorporating the travellers’ experience value in assessing the quality of transit nodes: A Rotterdam case study. Case Studies on Transport Policy.

[CR28] Haeri SAS, Rezaei J (2019). A grey-based green supplier selection model for uncertain environments. Journal of Cleaner Production.

[CR29] Hansson SO (2007). Philosophical problems in cost-benefit analysis. Economics and Philosophy.

[CR30] Hellström T (2003). Systemic innovation and risk: Technology assessment and the challenge of responsible innovation. Technology in Society.

[CR31] Huldtgren A (2015). Design for values in ICT information and communication technologies (handbook of ethics, values, and technological design: Sources, theory, values and application domains).

[CR32] Katz ML, Shapiro C (1986). Technology adoption in the presence of network externalities. The Journal of Political Economy.

[CR33] Kaufmann S, Künzel K, Loock M (2013). Customer value of smart metering: Explorative evidence from a choice-based conjoint study in Switzerland. Energy Policy.

[CR34] Kheybaria S, Kazemi M, Rezaeic J (2019). Bioethanol facility location selection using best-worst method. Applied Energy.

[CR35] King NJ, Jessen PW (2014). Smart metering systems and data sharing: Why getting a smart meter should also mean getting strong information privacy controls to manage data sharing. International Journal of Law and Information Technology.

[CR36] Kroes P, Van de Poel I, van den Hoven J, Vermaas PE, van de Poe I (2015). Design for values and the definition, specification, and operationalization of values. Handbook of ethics, values, and technological design.

[CR37] Ligtvoet A, Van de Kaa G, Fens T, Van Beers CP, Herder PM, Van den Hoven MJ, Janssen M, Wimmer MA, Deljoo A (2015). Value sensitive design of complex product systems. Policy practice and digital science: Integrating complex systems, social simulation and public administration in policy research.

[CR38] Linstone HA, Turoff M (1975). The delphi method.

[CR39] Liu HC, Quan MY, Li Z, Wang ZL (2019). A new integrated MCDM model for sustainable supplier selection under interval-valued intuitionistic uncertain linguistic environment. Information Sciences.

[CR40] Lo HW, Liou JJ, Wang HS, Tsai YS (2018). An integrated model for solving problems in green supplier selection and order allocation. Journal of Cleaner Production.

[CR41] McKenna E, Richardson I, Thomson M (2012). Smart meter data: Balancing consumer privacy concerns with legitimate applications. Energy Policy.

[CR42] Milchram C, Van de Kaa G, Doorn N, Künneke R (2018). Moral values as factors for social acceptance of smart grid technologies. Sustainability.

[CR43] Mitchell RK, Agle BR, Wood DJ (1997). Toward a theory of stakeholder identification and salience: Defining the principle of who and what really counts. Academy of Management Review.

[CR44] Mustajoki J, Hämäläinen RP, Salo A (2005). Decision support by interval SMART/SWING—Incorporating imprecision in the SMART and SWING methods. Decision Sciences.

[CR45] Nawaz F, Asadabadi MR, Janjua NK, Hussain OK, Chang E, Saberi M (2018). An MCDM method for cloud service selection using a Markov chain and the best-worst method. Knowledge-Based Systems.

[CR46] Nie RX, Tian ZP, Wang JQ, Zhang HY, Wang TL (2018). Water security sustainability evaluation: Applying a multistage decision support framework in industrial region. Journal of Cleaner Production.

[CR47] Omrani H, Alizadeh A, Emrouznejad A (2018). Finding the optimal combination of power plants alternatives: A multi response Taguchi-neural network using TOPSIS and fuzzy best-worst method. Journal of Cleaner Production.

[CR48] Onstein, A. T., Ektesaby, M., Rezaei, J., Tavasszy, L. A., & Van Damme, D. A. (2019). Importance of factors driving firms’ decisions on spatial distribution structures. *International Journal of Logistics Research and Applications* (in press).

[CR49] Oosterlaken I (2015). Applying value sensitive design (VSD) to wind turbines and wind parks: An exploration. Science and Engineering Ethics.

[CR50] Owen R, Bessant JR, Heintz M (2013). Responsible innovation: Managing the responsible emergence of science and innovation in society.

[CR51] Pesch U, Correljé A, Cuppen E, Taebi B, Van der Grift E, Asveld L, van Dam-Mieras R, Swierstra T, Lavrijssen S, Linse K, van den Hoven J (2017). Formal and informal assessment of energy technologies. Responsible innovation 3. A European agenda.

[CR52] Raz J (1986). The morality of freedom.

[CR53] Rezaei J (2015). Best-worst multi-criteria decision-making method. Omega.

[CR54] Rezaei J (2016). Best-worst multi-criteria decision-making method: Some properties and a linear model. Omega.

[CR55] Rezaei J, Kothadiya O, Tavasszy L, Kroesen M (2018). Quality assessment of airline baggage handling systems using SERVQUAL and BWM. Tourism Management.

[CR56] Rezaei J, Nispeling T, Sarkis J, Tavasszy L (2016). A supplier selection life cycle approach integrating traditional and environmental criteria using the best worst method. Journal of Cleaner Production.

[CR57] Rezaei J, Papakonstantinou A, Tavasszy L, Pesch U, Kana A (2019). Sustainable product-package design in a food supply chain: A multi-criteria life cycle approach. Packaging Technology and Science.

[CR58] Rezaei J, Van Roekel WS, Tavasszy L (2018). Measuring the relative importance of the logistics performance index indicators using Best Worst Method. Transport Policy.

[CR59] Rezaei J, Wang J, Tavasszy L (2015). Linking supplier development to supplier segmentation using Best Worst Method. Expert Systems with Applications.

[CR60] Rosenkopf L, Tushman ML (1998). The coevolution of community networks and technology: Lessons from the flight simulation industry. Industrial and Corporate Change.

[CR61] Saaty TL (1977). A scaling method for priorities in hierarchycal structures. Journal of Mathematical Psychology.

[CR62] Saaty TL (1990). Decision making for leaders: The analytic hierarchy process for decisions in a complex world.

[CR63] Salimi N (2017). Quality assessment of scientific outputs using the BWM. Scientometrics.

[CR64] Salimi N, Rezaei J (2018). Evaluating firms’ R&D performance using best worst method. Evaluation and Program Planning.

[CR65] Schilling MA (1998). Technological lockout: An integrative model of the economic and strategic factors driving technology success and failure. Academy of Management Review.

[CR66] Schilling MA (2002). Technology success and failure in winner-take-all markets: The impact of learning orientation, timing, and network externalities. Academy of Management Journal.

[CR67] Stilgoe J, Owen R, Macnaghten P (2013). Developing a framework for responsible innovation. Research Policy.

[CR68] Suarez FF (2004). Battles for technological dominance: An integrative framework. Research Policy.

[CR69] Taebi B (2017). Bridging the gap between social acceptance and ethical acceptability. Risk Analysis.

[CR70] Taebi B, Correljé A, Cuppen E, Dignum M, Pesch U (2014). Responsible innovation and an endorsement of public values: The need for interdisciplinary research. Journal of Responsible Innovation.

[CR71] Taebi B, Kadak AC (2010). Intergenerational considerations affecting the future of nuclear power: Equity as a framework for assessing fuel cycles. Risk Analysis.

[CR72] Taebi B, Kloosterman JL, Van den Hoven J, Vermaas P, Van de Poel I (2015). Design for values in nuclear technology. Handbook of ethics, values, and technological design.

[CR73] Tetlock PE (2003). Thinking the unthinkable: Sacred values and taboo cognitions. Trends in Cognitive Sciences.

[CR74] Umbrello S (2018). The moral psychology of value sensitive design: The methodological issues of moral intuitions for responsible innovation. Journal of Responsible Innovation.

[CR75] Umbrello S, De Bellis AF, Yampolskiy RV (2018). A value-sensitive design approach to intelligent agents. Artificial intelligence safety and security.

[CR76] Van de Kaa G, De Bruijn JA (2015). Platforms and incentives for consensus building on complex ICT systems: The development of WiFi. Telecommunication Policy.

[CR77] Van de Kaa G, De Vries HJ, Rezaei J (2014). Platform selection for complex systems: Building automation systems. Journal of Systems Science and Systems Engineering.

[CR78] van de Kaa G, Fens T, Rezaei J (2019). Residential grid storage technology battles: A multi-criteria analysis using BWM. Technology Analysis and Strategic Management.

[CR79] Van de Kaa, G., Fens, T., Rezaei, J., Kaynak, D., Hatun, Z., & Tsilimeni-Archangelidi, A. (2019b). Realizing smart meter connectivity: Analyzing the standards battle between power line communication, mobile telephony, and radio frequency using the Best Worst Method. *Renewable and Sustainable Energy Reviews*.

[CR80] Van de Kaa G, Janssen M, Rezaei J (2018). Standards battles for business-to-government data exchange: Identifying success factors for standard dominance using the Best Worst Method. Technological Forecasting and Social Change.

[CR81] Van de Kaa G, Van den Ende J, De Vries HJ (2015). Strategies in network industries: The importance of inter-organisational networks, complementary goods, and commitment. Technology Analysis and Strategic Management.

[CR82] Van de Kaa G, Van den Ende J, De Vries HJ, Van Heck E (2011). Factors for winning interface format battles: A review and synthesis of the literature. Technological Forecasting and Social Change.

[CR83] Van de Poel I, Meijer A (2009). Values in engineering design. Philosophy of technology and engineering sciences.

[CR84] Van de Poel I, Van den Hoven J, Vermaas P, Van de Poel I (2015). Conflicting values in design for values. Handbook of ethics, values, and technological design: Sources, theory, values and application domains.

[CR85] Van de Poel I (2015). Design for values in engineering (handbook of ethics, values, and technological design: Sources, theory, values and application domains).

[CR86] Van de Poel IR, Franssen M, Vermaas P, Kroes PA, Meijers AWM (2016). A coherentist view on the relation between social acceptance and moral acceptability of technology. Philosophy of technology after the empirical turn.

[CR87] Van den Hoven J (2005). Design for values and values for design. Information Age.

[CR88] Van den Hoven J (2007). ICT and value sensitive design.

[CR89] Van den Hoven J, Lokhorst GJ, Van de Poel I (2012). Engineering and the problem of moral overload. Science and Engineering Ethics.

[CR90] Van den Hoven J, Van de Poel I, Vermaas P (2015). Handbook of ethics and values in technological design.

[CR91] Van den Hoven J, Vermaas PE, Van de Poel I, Van den Hoven J, Vermaas P, Van de Poel I (2015). Design for values: An introduction. Handbook of ethics, values, and technological design: Sources, theory, values and application domains.

[CR92] Vermaas PE, Tan YH, van den Hoven J, Burgemeestre B, Hulstijn J (2010). Designing for trust: A case of value-sensitive design. Knowledge, Technology and Policy.

[CR93] Von Schomberg R (2011). Towards responsible research and innovation in the information and communication technologies and security technologies fields.

[CR94] Warnier M, Dechesne F, Brazier F, van den Hoven J, Vermaas PE, van de Poel I (2015). Design for the value of privacy. Handbook of ethics, values, and technological design: Sources, theory, values and application domains.

[CR95] Wüstenhagen R, Wolsink M, Burer MJ (2007). Social acceptance of renewable energy innovation: An introduction to the concept. Energy Policy.

[CR96] Wüstenhagen R, Wolsink M, Bürer MJ (2007). Social acceptance of renewable energy innovation: An introduction to the concept. Energy Policy.

[CR97] Zhao H, Guo S, Zhao H (2018). Selecting the optimal micro-grid planning program using a novel multi-criteria decision making model based on grey cumulative prospect theory. Energies.

[CR98] Zhou S, Brown MA (2017). Smart meter deployment in Europe: A comparative case study on the impacts of national policy schemes. Journal of Cleaner Production.

